# Supplementary material for: Learning curve and surgical outcome of robotic assisted colorectal surgery with ERAS program
Source: Sci Rep. 2022 Nov 29;12:20566. doi: 10.1038/s41598-022-24665-w (PMC9709162; doi:10.1038/s41598-022-24665-w)
Supplement: Supplementary file 1 — Supplementary Information 1. [file 41598_2022_24665_MOESM1_ESM.zip › LSC Raw data-Ñ[▒K/130/N_ñw╝╨░O▒Kñσ.pdf]

W52 / W52

臺中榮民總醫院
手術室護理記錄

[redacted]

基本資料

- 室別： 開刀房-06室，手術科別： CRS，手術類別： 常規，日期： 2021/07/15
- 進入時間： 1145 ； 麻醉開始時間： 1158 ； Time Out時間： 1255 ； 劃刀時間： 1256 ； 手術結束時間： 1725 送出時間： 1755 ；
- 麻醉方式： GE

  診斷： Malignant tumor of upper rectum

  手術名稱： Laparoscopic Anterior resection and anastomosis - malignant

  手術局部用藥： nil

  術前預防性抗生素：
  - 抗生素名稱一： Veterin 1GM IVD
  - 時間： 1245
  - 上次簽章: 廖子晴

  ，追加預防性抗生素：
- 手術醫師： 蔣鋒帆，楊曙榮，陳映晴，童冠愷
- 麻醉醫師： VS 吳志成
-
  - 刷手護士A ： 羅媗鈴，時間： 1145，刷手護士B ： 邱麗嫆，時間： 1200，接替刷手一： 許雅芳，時間： 1500
  - 流動護士A ： 許雅芳，時間： 1145，流動護士B ： 廖子晴，時間： 1200，接替流動一： 詹佳穎，時間： 1500，接替流動二： 劉雅汶，時間： 1730

皮膚狀況

A-V Shunt： 無

術前： 完整

記錄者： 上次簽章: 廖子晴

術後： 完整

記錄者： 上次簽章: 詹佳穎

手術準備

手術臥式 單一手術臥式

-
  - 手術臥式： 截石臥式
  - 使用附件： 溫毯 38 ℃，頭圈，腳架
  - 皮膚準備： 10%Aq Betadine，Hibiscrub，2% Chlorhexidine
  - 電燒： 使用電燒: 電極片位置： 其它： 迴路電擊墊，記錄者： 上次簽章: 廖子晴

止血帶T： 無

植入物

- 植入物名稱/號碼/數量/部位： EEA/33mm x 1 釘
  TA/45-4.8mm/Rectum

，記錄者： 上次簽章: 廖子晴

標本送檢

- 細菌培養 無
- 細胞學 無
- 冰凍切片 無
- 病理標本

  組織名稱一： IMA root

  ，

  組織名稱二： distal cut end

  ，

  組織名稱三： RECTUM

- 截肢 無

標本交付 標本： 2 袋 , 標本： 0 盒 , 標本： 1 瓶 , 細胞學： 0 管 , Culture Tube： 0 管 , 被交付醫師： CR 楊曙榮 ,

- 記錄者： 上次簽章: 詹佳穎
- 時間： 1520

傷口術後敷料

傷口敷料： 傷口敷料： 無

- 請於圖上標示部位：

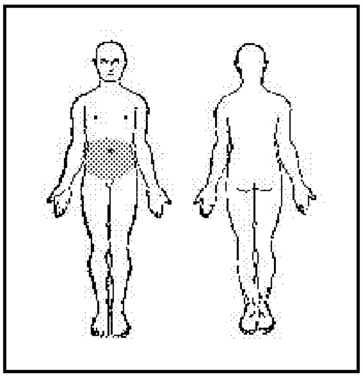

- 傷口敷料： 白紗

導管

導管	天數	導管資訊	插入日期	插入時間	更換日期	拔除日期	拔除時間	拔除原因	預計拔除/更換	預計拔除/更換日期
NG Tube(1)	1	號碼:16 Fr.; 部位:胃;	2021/07/15	1215						
Penrose Drain(1)	1	號碼:12 mm.; 部位:骨盆腔;	2021/07/15	1625						
Foley(1)	1	號碼:16 Fr.; 部位:膀胱; 註記:balloon10c.c;	2021/07/15	1225						
Double Lumen CVC(1)	1	號碼:8 Fr.; 深度:15cm; 部位:jugular (右); 象限:RU;	2021/07/15	1220						

用物計數

- 手術中出血量： 150 c.c.
- 手術中尿量： 600 c.c.
-
    - 其它： 無

用物計數： 單一切口

- 1.器械： 正確 , 2.縫針： 正確 , 3.刀片： 正確 , 4.紗布： 正確 , 5.腹部墊： 正確 , 6.紗球： 未使用 , 7.PEANUTS ： 未使用 , 8.紗條： 未使用 , 9.絲帶： 未使用 , 10.針頭： 未使用 , 11.Vessel loop： 未使用 , 12.Boots： 未使用 , 13.Jelco： 未使用 , 14.棉條： 未使用 ,
    - 第一次：
        - 刷手護士 ： 許雅芳
        - 流動護士： 上次簽章: 詹佳穎
        - 時間： 1550

  ,
    - 第二次：
        - 刷手護士 ： 許雅芳
        - 流動護士： 上次簽章: 詹佳穎
        - 時間： 1625

  ,
    - 第三次：
        - 刷手護士 ： 許雅芳
        - 流動護士： 上次簽章: 詹佳穎
        - 時間： 1650

備註

備註： NIL

- 記錄者： 上次簽章: 廖子晴
- 時間： 1255

填寫護理人員 劉雅汶

正式摘要

	W52 / W52	

doctorRank:B
